# A Model generation method and iteration algorithm for optimising fire protection thickness

**DOI:** 10.1016/j.mex.2024.102632

**Published:** 2024-02-24

**Authors:** Yang Li, Zhuoran Feng, Simon Thurlbeck, Meini Su

**Affiliations:** Department of Solids and Structures, The University of Manchester, Booth St E, Manchester M13, UK

**Keywords:** Heat transfer, Abaqus kernal scripting method, Intumescent coating, Fire safety design, Model generation method for composite model and Iteration algorithm for optimising fire protection thickness

## Abstract

With temperatures rising above 1000 °C within 5 min, hydrocarbon fire causes rapid strength degradation of structural steel members. It is among the most dangerous hazards, such as boiling liquid expanding vapour explosion (BLEVE) in the oil and gas industry. Intumescent coating as passive protection is widely adopted to prevent the steel structure from material property reduction. However, when optimising fire protection with heat transfer simulation, repetitive modelling work and lacking recalculation principle hinder productivity improvement. This method is developed to generate steel beam models and provides an effective algorithm to optimise coating thickness considering the temperature of a specific region. The main functions of the method include:

• Providing section dimensions, initial insulation thickness, target temperature and heating time, temperature allowance and mesh size as variables.
• Automatically generating the Abaqus steel beam model under 3-side heating conditions.
• Effective iteration algorithm to modify fire protection thickness: test containing 38 Universal beam sections with a 5 °C allowance below target shows that 55.2% were completed within five iterations and 76.3% were completed within eight iterations.

## Graphical abstract

**Figure:**
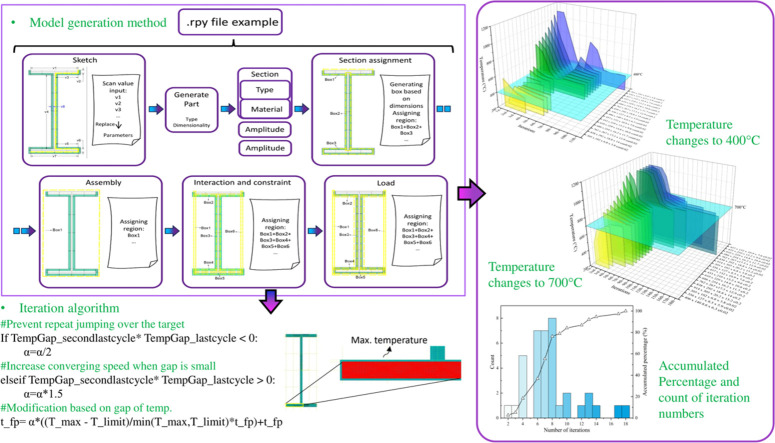

Specifications table

**Table:** 

Subject area:	Engineering
More specific subject area:	*Fire safety design*
Name of your method:	Model generation method for composite model and Iteration algorithm for optimising fire protection thickness
Name and reference of original method:	ABAQUS kernel scripting method Dassault Systems, Scripting User's Manual. 2013, Simulia Corp. Dassault Systems, Theory Manual. 2013, Simulia Corp.
Resource availability:	https://github.com/Supernova772/Abaqus-Tools/blob/main/Iterative%20algorithm%20hydrocarbonfire.py https://github.com/Supernova772/Test-data-of-iteration-algorith, https://github.com/Supernova772/Test-data-of-iteration-algorithm/blob/main/Test_record.txt

**Method details**

**Notations**

d Section depth

t_target_ Target time

T_limit_ Limiting temperature

T_max_ Maximum temperature

σT Temperature allowance: σT ≥ T_limit_T_max_

w_fp_ Fire protection thickness

Alt-text: Unlabelled box

## Introduction

In addition to the commonly used graphic user interface (GUI), Abaqus offers a Python-based kernel scripting method for researchers and engineers to perform extensive operations not incorporated in the ABAQUS. With the support of Python community resources, developments on both pre and post-analysis modules of ABAQUS are conveniently executed.

The Abaqus scripting interface uses the object-oriented Python scripting language. The kernel interprets the commands to create the internal representations [1]. It provides a comprehensive list of Abaqus commands. These commands allow users to automatically perform repetitive tasks, carry out parametric studies and modify model databases created in GUI et al. Additionally, the commands performed in GUI are recorded to the .rpy file as a helpful reference. William [2] adopted the Abaqus python scripting method to generate simulation models of the topologically interlocked material systems. Tang et al. proposed an approach to calculate the shake limit of geo-structure by adding a returning mapping scheme in the direct solution method [3]. Mengoni [4] developed a Python library opti4Aba to tune the model material to output the designated curve. An iterative optimisation algorithm to minimise the shape recovery in the sheet metal l-bending process is developed by Faiez et al. [5] by coupling the Abaqus/standard code and Python script. In the industry practice of fire protection design, optimising the fire thickness of the steel beam is largely based on the estimation of engineers. The development of numerical simulation has primarily improved the precision of design and provides an efficient tool for engineers. Moreover, the optimisation of fire protection through numerical simulation is based on trial and error, and it is time-consuming due to repetitiveness and lack of recalculation principle. On the other hand, when performing a parametric study with ABAQUS using the rpy. file, the analysis could not treat the models connected by constraints due to the machine code in the selection. To resolve these problems, a fully parametric method for generating a composite model and optimising steel temperature to a target temperature is developed. This optimisation algorithm remarkably reduces the laborious work through the automated process and improves the precision with numerical simulation by the optimisation algorithm. The fidelity of this method is then proved by a series of tests to extensive typical steel shapes.

## General process

**Fig. 1:**
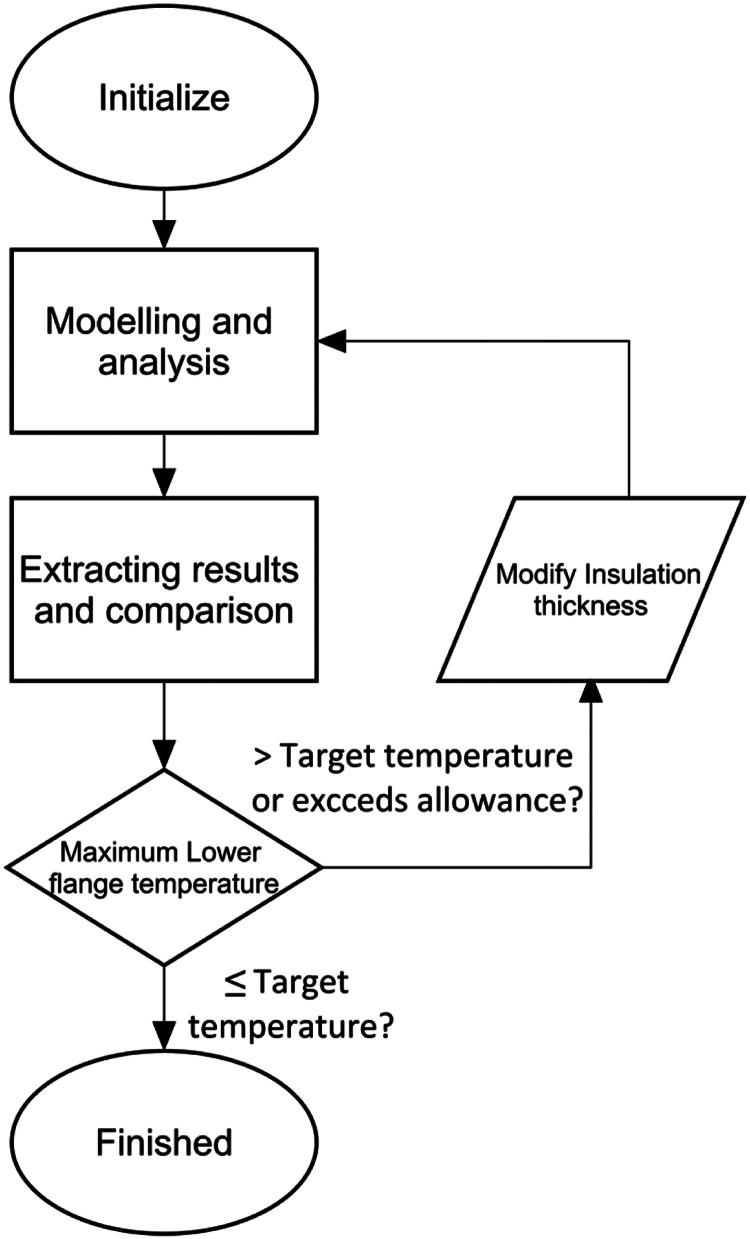
Modification process.

**Fig. 2:**
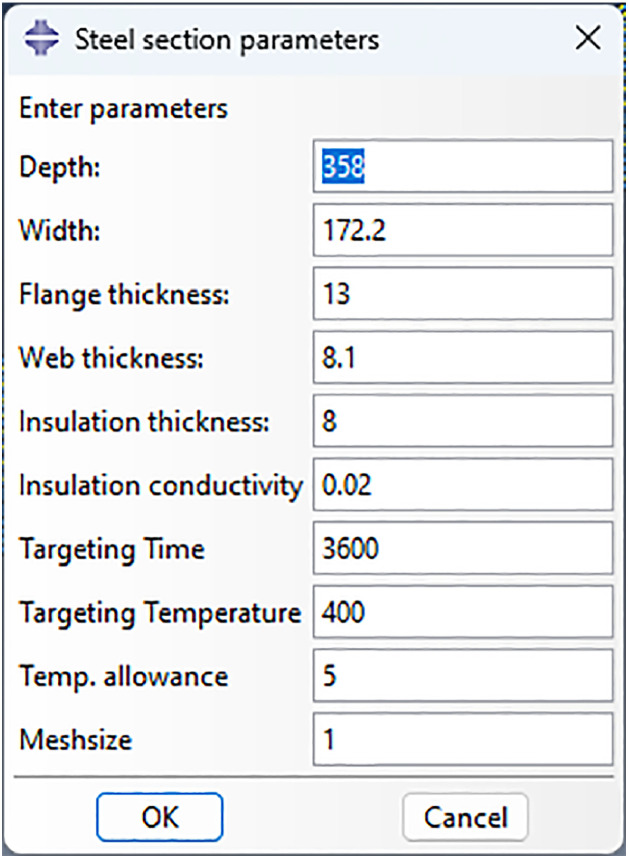
Parameters input interface.

To illustrate how the program works, the general optimisation process is shown in the flow chart below (Fig. 1). For scripting language, the program is written in the Python-based Abaqus scripting interface [[6], [7]] and Numpy [8] library is adopted for storing the data. Overall, the optimisation process contains three or four parts. The first part is establishing the composite model where the input values in rpy. file is replaced by the variables to transfer the parameters of interface (Fig. 2) and functions of contacting edges are written to replace the box selection in GUI. The second portion extracts the lower flange temperatures included in an element set from the .odb file through scripts, and the maximum value is stored for comparison. The third part compares it with the target temperature, and the optimisation algorithm recalculates the fire protection thickness through the simplified Newton method if the temperature is not satisfied. Lastly, the new model is established with new insulation thickness and submitted for analysis. The detail of each parts are presented in the sections below.

## Model generation method

**Fig. 3:**
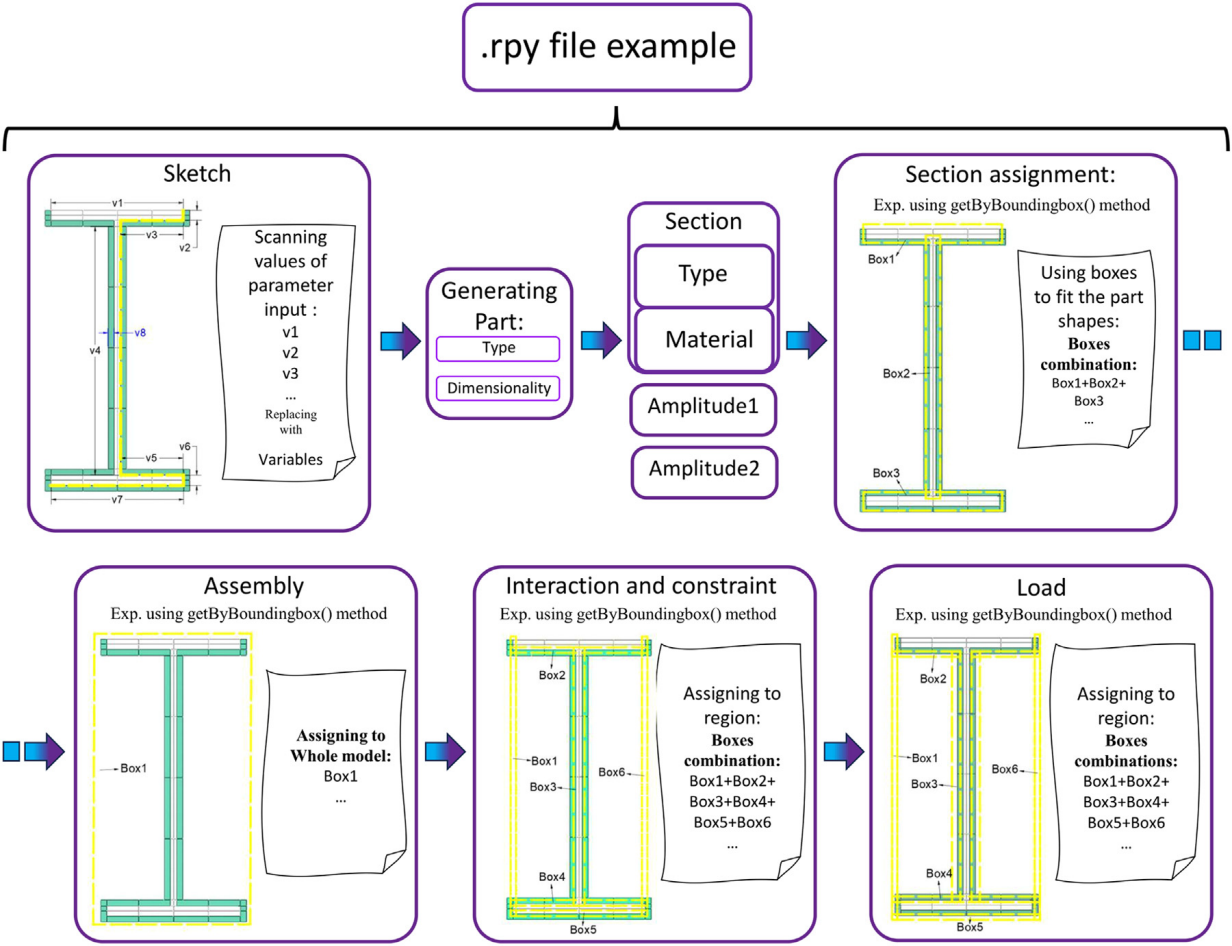
Replacing parameters and establishing interactions.

Although the .rpy file records the commands in the graphical user interface as scripts, machine code which is a variable changing with the model parameters in the getSequenceFromMask() method could not be easily interpreted. To resolve this problem, other approaches with more explicit variables, such as getClosest(), getByBoundingBox() and getByBoundingCylinder() in the scripting interface could be adopted to replace its function. A flowchart using the kernel scripting method to produce model with similar geometry properties is shown in Fig. 3.

The models to optimise are a set of 3-side protection steel beams established with heat transfer elements. The modelling scripts are generated following the methods in the ABAQUS scripting reference [6]. For modelling parameters, the variables include section dimensions, insulation thickness and conductivity, mesh size, lower flange target temperature, and allowance of temperature residual by replacing original inputs with variables (Fig. 2). The getByBoundingBox() method is used to establish the region for interaction, and a 0.01 mm allowance of the selection is added to prevent errors. This method requires at least one of the maximum or minimum values of XYZ coordinates, and it should be noted that including unconnected edges in the box could reduce the number of edge arrays. The same method establishes tie constraints between steel and fire protection. The process of establishing models is defined as a function for reuse. For the analysis steps definition, the total time was replaced with the variable of target time for computational efficiency. To make the mesh uniform in geometry, the steel and fire protection at the junction was partition face which adopted the scripts generated by the GUI.

**Fig. 4:**
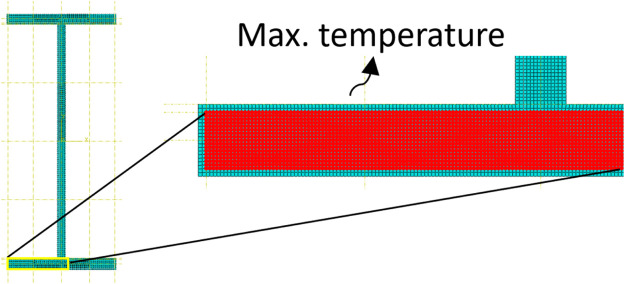
Schematics of mesh and temperature extraction.

For analysis flexibility, the mesh size is set as a variable to fit the section dimension. A Node set is established by the getByBoundingBox() method to output the temperatures (Fig. 4). The degree of freedom of temperature for the solid section is NT11 in ABAQUS. After that, the model is submitted and waits for completion. In addition, the time.sleep method between heavy computation processes such as mesh generation to prevent jams, and it could also be used to refresh messages in the terminal.

## Extraction of temperatures and comparison

The target temperature is set as the maximum temperature of the lower flange. The xyDataListfromfield method is adopted for outputting temperature. This method creates a list object with three layers of indexes. Taking the [0][*i*][0] for example, the first index 0 denotes data output, the second index *i* is the time frame number, and the third index is for step time. The temperature is only extracted before the target time, and a timeframe object is used to set up a frame repository. The getSubset() method derives the temperature object of all the nodals of the lower flange. To get the maximum temperature value, the ‘for’ cycle is utilised to store the object.data to a list and get the maximum value. Modifying the insulation thickness requires the maximum temperature to be higher than the target temperature or lower than T_limit_ – σT.

## Iterative optimisation algorithm

The modification algorithm contains three parts for different purposes. The first part is the general function to modify the insulation thickness based on the gap between the simulation and the targeting temperature. The second part increases the speed of convergency when the temperature is close to the target and reduces the thickness change when the simulation temperature jumps over the target. Lastly, the third part is setting the barrier to prevent thickness error.

Specifically, in the first part, the modification algorithm is wfp = α* (T_max_ - T_limit_)/ min(T_max_, Tlimit)* wfp + wfp, where α is the modifying coefficient of the fire protection thickness increment. The second part of modifying the α runs before the first part. Specifically, if the last two (Tmax-Tlimit) are the same sign, the α multiplies 1.5 for the next wfp. And if the previous two (Tmax-Tlimit)s have opposite signs, the α divides 2 for the next iteration.

The third part sets the lower and upper limits of fire protection thickness between 0.1 mm and half the interval between the upper and lower flange (Table 1). The script of the iteration algorithm in Python is shown in Table 1.

**Table 1:** Scripts of the optimisation algorithm.

# Stacking the temperature gaps of the last two cycles TempGap = [0,0] def iteration_algorithm(w_fp,T_max,T_limit,TempGap,α): # Discarding the second-last temperature gap TempGap.pop(0) round(T_max,2) *a* = T_max-T_limit # Appending the last temperature gap TempGap.append(a) # Preventing jumping repeatly over the target if TempGap [0]* TempGap [1] < 0: α=α/2 # Increasing converging speed when the gap is small elif TempGap [0]* TempGap [1] > 0: α=α*1.5 # Modifying fire protection thickness based on the gap of temperature w_fp=α*((T_max-T_limit)/min(T_max,T_limit)*w_fp)+*w*_fp w_fp=round(w_fp,2) if w_fp <= 0: w_fp=0.1 elif w_fp >= 0.5*(d-2*f_t): w_fp= 0.5*(d-2*f_t)−0.01 return w_fp,α

## Optimisation results and algorithm effectiveness

**Fig. 5:**
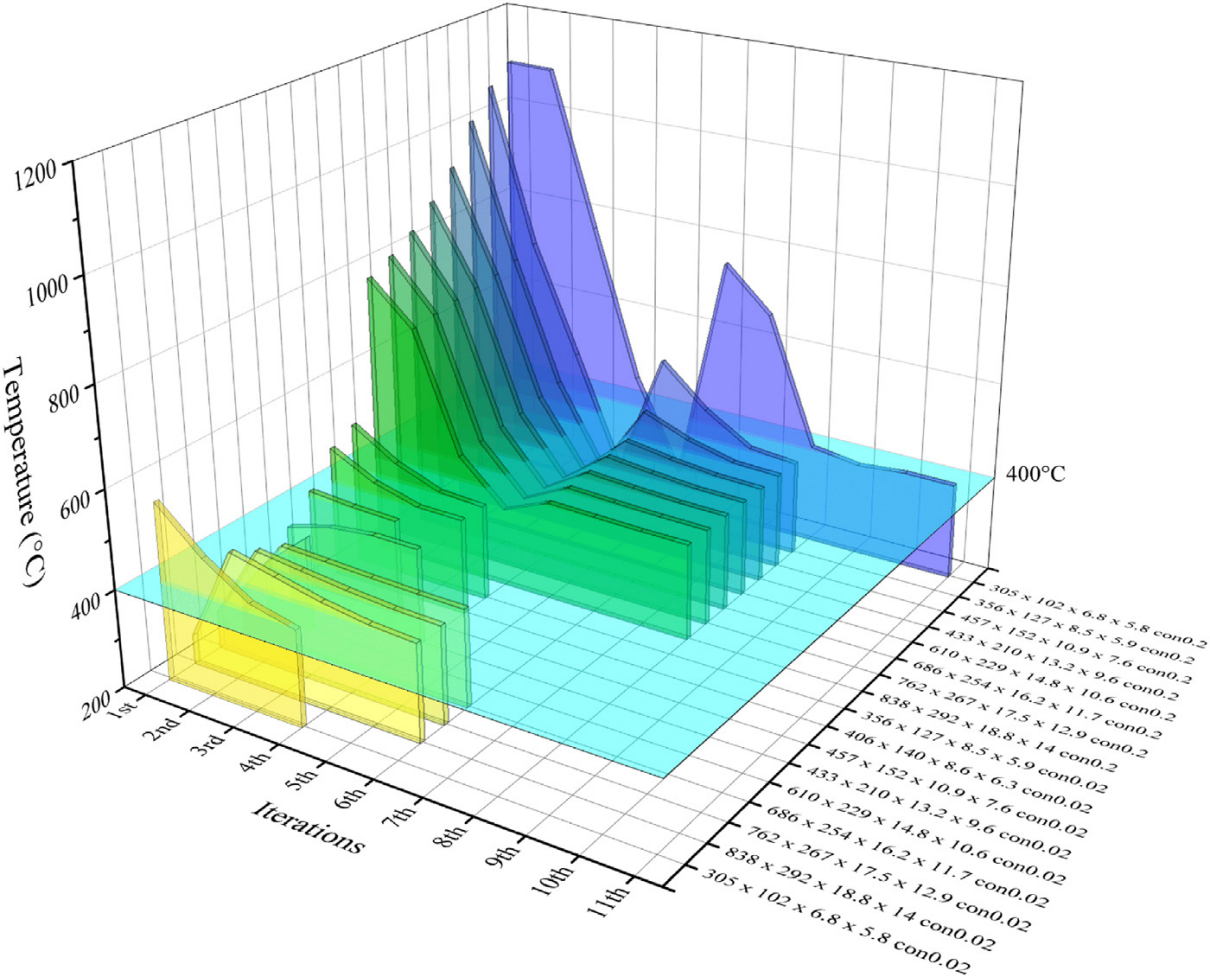
Temperature change during optimisation to 400 °C.

**Fig. 6:**
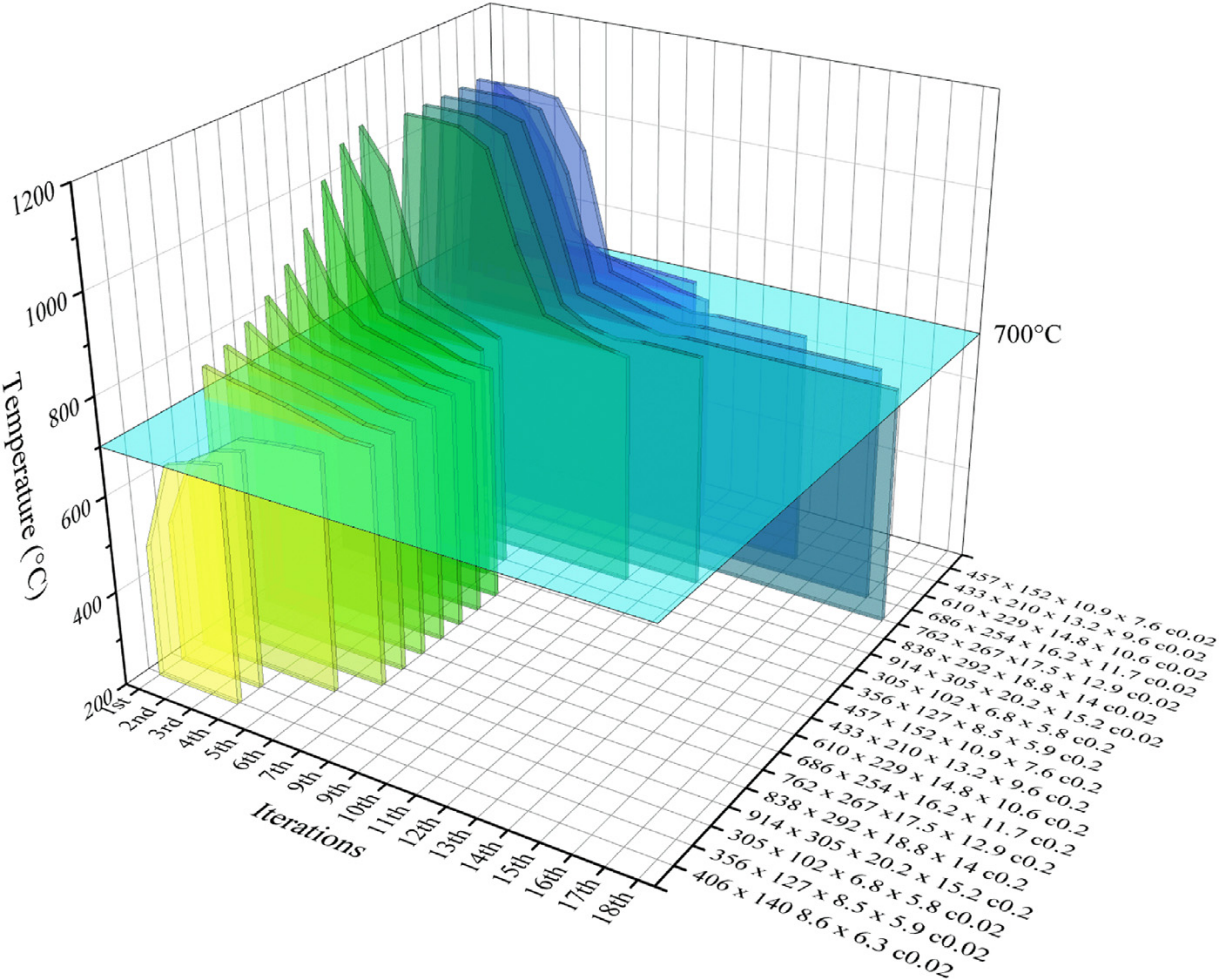
Temperature change during optimisation to 700 °C.

The present tests involve changes in insulation thickness, conductivity and target temperature. A total of 38 tests were carried out, and Table 1, Table 2. presents the variations in insulation thickness and temperature of representative sections during the process. The algorithm utilised in this study completes the process for a UB457 × 152 × 10.9 × 7.6 section with an initial temperature of 1009.1 °C within eight iterations. Notably, the increments of insulation decrease as the temperature approaches the target value. Fig. 5, Fig. 6 show the effect of the algorithm on extensive sections. For the numbering system, the con/c indicates insulation conductivity in W/m*K. In addition to reducing the temperature gap within moderate number of iterations. The algorithm effectively controlled the temperature from repeatedly jumping over the target value as shown in UB356 × 127 × 8.5 × 5.9 con0.2, UB 457 × 152 × 10.9 × 7.6 con0.2, UB838 × 292 × 13.8 × 14 con0.02 (Fig. 5) and UB610 × 229 × 14.8 × 10.6 c0.02, UB686 × 254 × 16.2 × 11.7 c0.02, UB762 × 267 × 17.5 × 12.9 c0.02 (Fig. 6).

**Table 2:** Insulation thickness and maximum temperature of representative sections.

Section dimensions		
Number of iterations	Fire protection thickness (mm)	Maximum temperature ( °C)
UB305 × 102 × 6.8 × 5.8 con0.02		
1	8	561.5
2	11.26	478.1
3	14.59	418.1
4	16.09	396.3
UB356 × 127 × 8.5 × 5.9 con0.02		
1	8	497.1
2	9.96	439.1
3	11.43	403.9
4	11.69	398.3
UB406 × 140 × 8.6 × 6.3 con0.02		
1	8	473.4
2	9.48	426.1
3	10.42	400.6
4	10.46	399.6
UB457 × 152 × 10.9 × 7.6 con0.02		
1	8	1009.1
2	20.3	729.4
3	45.63	480.7
4	66.55	319.3
5	47.44	464.2
6	51.77	426.9
7	54.75	402.9
8	55.27	398.8
UB433 × 210 × 13.2 × 9.6 con0.02		
1	8	361.96
2	7.15	389.28
3–11	6.85	400.02
12	6.86	399.65
UB610 × 229 × 14.8 × 10.6 con0.02		
1	8	315.75
2	5.84	396.59
UB686 × 254 × 16.2 × 11.7 con0.02		
1	8	291.96
2	5.01	411.19
3	5.08	407.35
4	5.15	403.58
5	5.2	400.92
6	5.22	399.87
UB762 × 267 × 17.5 × 12.9 con0.2		
1	8	806.09
2	9.22	765.62
3	10.53	748.6
4	12.19	735.64
5	14.31	720.83
6	16.49	701.75
7	16.81	698.03

**Fig. 7:**
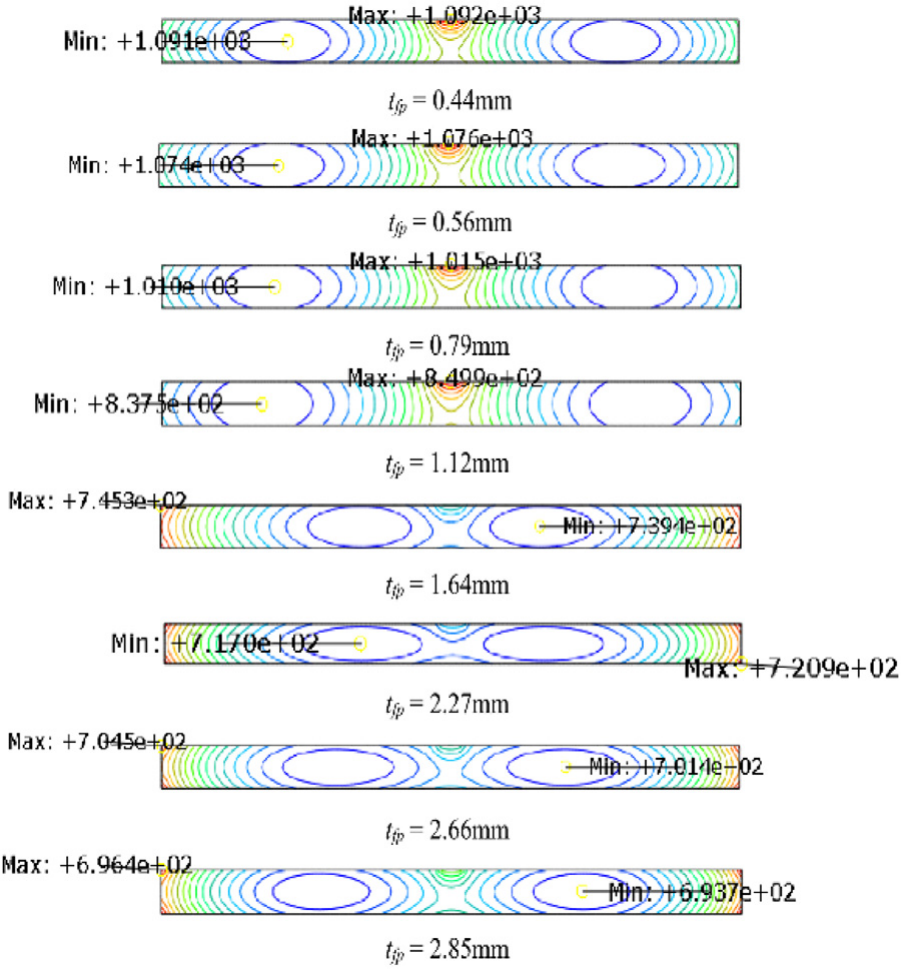
Temperature distribution of lower flange in test.

**Fig. 8:**
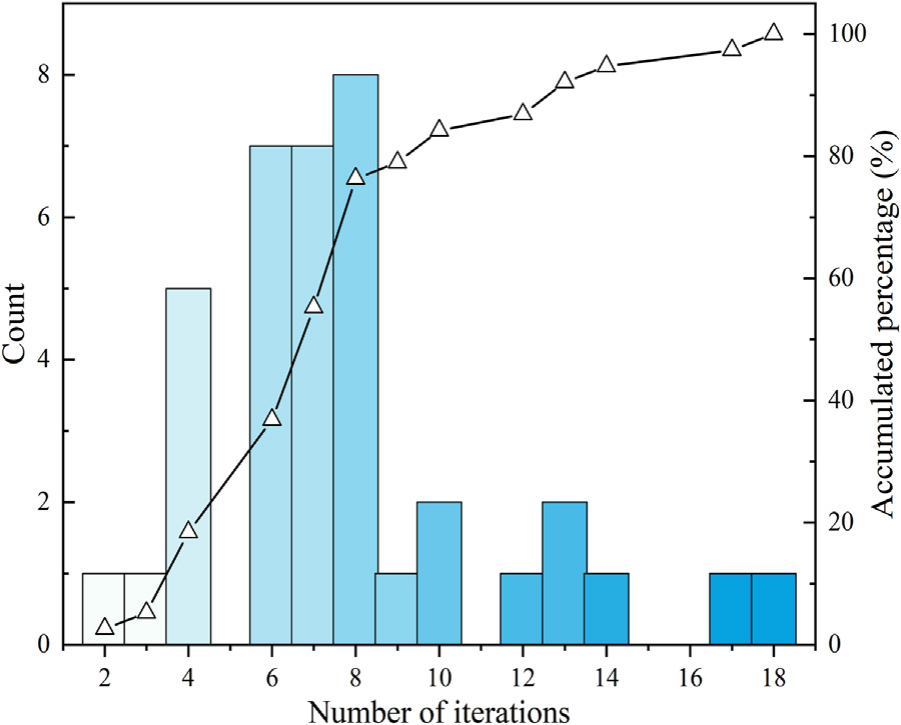
The proportion of optimisations of maximum iteration.

In the contour diagram (Fig. 7), a section with a 700°C steel target temperature in the lower flange is presented. The success to control the maximum temperatures in different temperature distributions proves the necessity to induce an inclusive node set in temperature records. For 55.2% of the tested validations, the optimisations are finished within seven iterations, and 76.3% are within eight iterations (Fig. 8). During this process, no artificial efforts are required, which significantly improves productivity.

## Conclusions

An iteration algorithm to modify the fire protection thickness and a heat transfer model generation method for the iteration process was established with Python-based Abaqus script. The target time, limiting temperature, temperature allowance, and mesh size are available for various circumstances. Validation on a wide range of sections with different initial temperatures shows that it performs well under various circumstances:

1. For 55.2% of the validation conducted, the algorithm finished optimisation within seven iterations. And 76.3% of the validations are completed within eight iterations.
2. All the testing optimisation processes of steel beam temperature converge within 16 iterations. In this process, no artificial effort is needed which significantly improves productivity without labour input.

For the prospective investigations, the adjusting coefficient α could be calibrated to increase the convergency rate. This method also applies to various iterative optimisation tasks.

## CRediT authorship contribution statement

**Yang Li:** Conceptualization, Methodology, Software, Writing – original draft, Writing – review & editing. **Zhuoran Feng:** Methodology, Supervision, Funding acquisition. **Simon Thurlbeck:** Methodology, Supervision, Funding acquisition. **Meini Su:** Methodology, Supervision.

## Declaration of competing interest

The authors declare that they have no known competing financial interests or personal relationships that could have appeared to influence the work reported in this paper.

## Data Availability

The raw scripts and test data are avalible in the address below. https://github.com/Supernova772/Abaqus-Tools/blob/main/Iterative%20algorithm%20hydrocarbonfire.py and Test-data-of-iteration-algorithm.
